# Importance of prefrontal meta control in human-like reinforcement learning

**DOI:** 10.3389/fncom.2022.1060101

**Published:** 2022-12-21

**Authors:** Jee Hang Lee, Joel Z. Leibo, Su Jin An, Sang Wan Lee

**Affiliations:** ^1^Department of Human-Centered Artificial Intelligence, Sangmyung University, Seoul, South Korea; ^2^Google DeepMind, London, United Kingdom; ^3^Department of Bio and Brain Engineering, Korea Advanced Institute of Science and Technology, Daejeon, South Korea; ^4^Program of Brain and Cognitive Engineering, Korea Advanced Institute of Science and Technology, Daejeon, South Korea; ^5^KAIST Center for Neuroscience-Inspired Artificial Intelligence, Korea Advanced Institute of Science and Technology, Daejeon, South Korea; ^6^KAIST Institute for Health Science and Technology, Korea Advanced Institute of Science and Technology, Daejeon, South Korea; ^7^KAIST Institute for Artificial Intelligence, Korea Advanced Institute of Science and Technology, Daejeon, South Korea

**Keywords:** reinforcement learning, neuroscience of RL, prefrontal meta control, model-based and model-free RL, human-aligned RL models

## Abstract

Recent investigation on reinforcement learning (RL) has demonstrated considerable flexibility in dealing with various problems. However, such models often experience difficulty learning seemingly easy tasks for humans. To reconcile the discrepancy, our paper is focused on the computational benefits of the brain's RL. We examine the brain's ability to combine complementary learning strategies to resolve the trade-off between prediction performance, computational costs, and time constraints. The complex need for task performance created by a volatile and/or multi-agent environment motivates the brain to continually explore an ideal combination of multiple strategies, called meta-control. Understanding these functions would allow us to build human-aligned RL models.

## 1. Introduction

Suppose a new game was released. Reading the detailed “how to play” instructions would be a desirable first step toward mastering the game. However, when such instruction is not offered, we might consider playing the game without prior knowledge. This undirected approach often works in practice. In fact, it is often the case that we can understand the gist of the game after only a few trials, including its rules, goals, and environmental structure, and how to draw up a draft strategy before deliberate planning. Subsequent experience brings further improvement from such initial information, rapidly refining a strategy suitable to various contexts and goals.

This example briefly describes the human capability of forming optimal behaviors based on learning from experiences, the so-called theory of reinforcement learning (RL) in the human mind. Inspired by the psychology and neuroscience of such human behavior, RL theory provided the mathematical scaffolding to describe how humans learn from past experiences (Schultz, 1998; Sutton and Barto, 1998). Due to breakthroughs in deep learning and a steep increase in computing power, RL theory and practice have led to remarkable advances in our ability to design artificial agents with super-human performance. Results demonstrate the applicability of RL in various domains, including games (Mnih et al., 2015; Silver et al., 2016, 2017a,b, 2018; Schrittwieser et al., 2020), large-scale Markov decision problems (Sutton and Barto, 1998; Szepesvári, 2010; Sigaud and Buffet, 2013; Covington et al., 2016; Evans and Gao, 2016; Dulac-Arnold et al., 2021), and non-linear and stochastic optimal control problems without explicit representations of environments (Bertsekas and Tsitsiklis, 1995; Si, 2004; Busoniu et al., 2010; Fujimoto et al., 2019; Vecerik et al., 2019).

Nevertheless, the computational principles underpinning RL algorithms still differ from the manner in which human RL works. The goal of RL is to develop a policy that specifies the choice of action for each state of the world so as to maximize the expected amount of future reward (Sutton and Barto, 1998). It is common to divide the space of principles supporting RL algorithms into two categories called, respectively, model-free (MF) and model-based (MB). Most state-of-the-art RL agents only incorporate the model-free (MF) RL principle (Doya et al., 2002; Daw et al., 2005), whereas human brains employ both MF and MB principles simultaneously (Dolan and Dayan, 2013).

In animals, MF RL is guided by the dopaminergic striatal system (Montague et al., 1996; Schultz et al., 1997). Based on the “trial-and-error” concept, MF RL incrementally updates the values of actions based on reward prediction error, which is the quantity that represents the discrepancy between the agent's prediction regarding reward and the actual rewards it receives from the environment. Iterating this process is a way to improve a policy, continually adjusting it to obtain more rewards until convergence. Many repetitions of a contingency are usually required to incorporate it into a policy by MF RL. As a result, policies learned by MF RL are said to be habit-like and automatic, making it harder to respond quickly to changing context. For instance, when there is a sudden change in the environment, or when a new goal is established, a major number of classical MF RL agents is likely to require re-learning to adapt to the change. This is often time-consuming and computationally inefficient. In particular, it requires a massive amount of new experience, since the effect of old information will only slowly decay by averaging as more and more contradictory information comes in later.

Humans can learn to adapt to environmental changes with a small amount (or an almost absence) of experience. As illustrated by the game example, human brains can go beyond the MF RL strategy to achieve impressive scores with high efficiency, speed, and flexibility in learning and behavior control—whether the game is entirely new or not (Lake et al., 2017). Recent progress in decision neuroscience indicates that the human brain also employs another learning strategy besides MF RL, called model-based (MB) RL (Daw et al., 2005; Dolan and Dayan, 2013). Using this strategy, the brain learns about different structures in the world, such as a state space or reward structure, leading to a deliberate behavioral policy that is sensitive to changes in the structure of the state space or goals (Kuvayev and Sutton, 1996; Doya et al., 2002; Daw et al., 2005; O'Doherty et al., 2015). In addition, humans have the ability to learn from a small number of observations: neural computations underlying rapid learning comprising so-called one-shot inference, can be distinguished from those used for incremental learning (Daw et al., 2006; Boorman et al., 2009; Badre et al., 2012; Lee et al., 2015; Meyniel et al., 2015). One-shot inference refers to the situation in which an agent learns rapidly from only a single pairing of a stimulus and a consequence, as often required for goal-driven choices. Incremental learning refers to the situation in which an agent gradually acquires new knowledge through “trial and error,” as seen in MF RL. A proper combination of these two types of learning may guide optimal behavior control in various situations within a relatively short time with access to a relatively small set of experiences (or data) in practice.

A neural mechanism called “meta-control” on top of multiple systems utilized for human RL accounts for behavioral flexibility, memory efficiency, and rapid learning speed in humans. Recent neuroimaging studies and the computational modeling used in RL studies have identified not only the respective neural correlates of MF and MB RL (Gläscher et al., 2010), but also the neuro-anatomical circuits responsible for arbitration between the two types of RL. The above studies are based upon the proposal that the arbitration is governed by the relative amount of uncertainty in the estimates of the two systems (Lee et al., 2014). Another meta-control ability is that of determining when to learn incrementally or rapidly to make inferences regarding the state of the world (learning from a few observations). This meta-control process can be extremely useful in guiding behavior during learning (Meyniel et al., 2015), in deciding whether to explore a new alternative or to pursue the currently available option (Daw et al., 2006; Badre et al., 2012), and in evaluating an alternative course of action (Boorman et al., 2009). It is noted that learning regarding the state-space is necessary for MB RL because the probabilistic representation of the state-space is an essential component required for computing the expected amount of future reward.

Understanding how the human brain implements these abilities, which state-of-the-art RL algorithms do not possess, would help us improve the design of RL algorithms as follows. First, an agent with an MB RL strategy would learn about the model of the environment and leverage this knowledge to guide goal-driven behavior. This includes action planning to foresee future episodes, even if they are computationally expensive, based on the model of the environment that the RL agent has in mind. Second, we expect that an RL agent with a rapid inference ability would learn a model of its environment from a very small number of samples, expediting the MB RL process. Finally, adaptive control of these functions serving RL, dubbed meta-control of RL, would resolve the trade-offs among prediction performance, computational load, and training efficiency.

This paper is organized as follows. In Section 2, we briefly overview the computational principles of RL and explore a few situations posing significant challenges to recent RL algorithms. We then discuss how the brain solves these challenges in a point-by-point manner in Section 3. We will specifically discuss MB and MF RL, one-shot inference in MB RL, and the meta-control process over these strategies. This discussion leads us to potential research questions presented in Section 4. A concluding remark is provided in Section 5.

## 2. Reinforcement learning—Basic ideas and challenges

### 2.1. Basic concepts

The theory of RL is a normative framework to account for the general principle describing how value-based, sequential decision-making takes place in humans (Mnih et al., 2015). RL algorithms in computer science are usually based on Markov Decision Processes (MDPs) (Bellman, 1957), which commonly model various sequential decision problems incorporating uncertainty in the environment.

Sequential choices, which occur in a range of real-world problems, is a fundamental task that any intelligent agents (including humans and animals) encounter in extended actions/interactions with their environment (Littman, 1996). In this circumstance, the agents iteratively try to make an optimal decision to achieve a goal in a sequential manner, through learning and inference. The agents need to act on what they have learned, use them to infer the decision which can possibly bring about the best outcomes, and learns from the obtained outcome for decision-making in the future. With this aim in mind, agents are capable of dealing with these sequential decision problems by means of *programming, search and planning*, or *learning* approaches. In general, agents who learn to make optimal decisions take into consideration a combinatorial approach—they carry out the *planning* in order to establish long-term actions in uncertain domains on the foundation of the *learning* about the environment. Sometimes the choice between exploiting what they already know and exploring new options that may lead to better outcomes (or worse) takes place for the purpose that either maximizing the effect of actions or toward a higher learning performance assuring a better model of an environment enabling the better outcomes (van Otterlo and Wiering, 2012).

These sequential decision problems are usually solved either by learning and planning given a model of the MDP referred to as MB RL, or by learning through actions/interaction with an unknown MDP referred to as MF RL. During the process, the desirability or undesirability of actions that agents choose in each state, and their effects are evaluated by a reward codified in a single scalar objective function. The objective of the agents is then the maximization of the (discounted) expected sum of the scalar reward at each step over time (Roijers et al., 2013).

A solution to the MDP is characterized by the Bellman optimality equation (Sutton and Barto, 1998).


(1) $$\begin{array}{l} Q^{*}(s,a)=E_{\left(s,a,s^{\prime }\right)}[R+\gamma \underset{a^{\prime }}{\max}Q^{*}(s^{\prime },a^{\prime })]\\ \;=\sum_{s^{\prime }}P(s,a,s^{\prime })(R+\gamma \underset{a^{\prime }}{\max}Q^{*}(s^{\prime },a^{\prime })) \end{array}$$

where the tuple 〈*s, a, s*′〉 refers to the current state *s*, an action *a*, and the state in the next time step *s*′, and *Q*(*s, a*) refers to the state-action value. *P*(*s, a, s*′) and *R* refer to the state-action-state transition probability and an immediate reward, respectively. It specifies that the value estimate for states and actions is based on the expectation over a state-space distribution of the quantity consisting of the amount of immediate reward plus the value estimate of the possible next state.

The goal of RL is to learn an optimal policy by estimating the expected amount of reward for each state or action Q^*^(*s, a*). Classical RL agents have employed various iterative methods (Sutton, 1988; Watkins, 1989; Barto and Duff, 1994; Singh and Sutton, 1996), but learning exact representations of value functions in high dimensional state space is often computationally intractable. In recent deep reinforcement learning research, non-linear, parameterized function approximation techniques are used to represent value functions, policies, and models of the environment. The combination of RL with deep learning has led to rapid advances in RL algorithm design with outstanding performance in many applications, including games, robot control and simulated environments (Silver et al., 2014; Lillicrap et al., 2015; Mnih et al., 2015, 2016; Van Hasselt et al., 2016; Kalashnikov et al., 2018; OpenAI, 2018; Vecerik et al., 2019). Mounting evidence suggests that similar algorithms are present in the mammalian brain and are embedded in different types of human decision-making systems (Daw et al., 2005; Dayan and Daw, 2008; Rangel et al., 2008; Balleine and O'doherty, 2010; Dolan and Dayan, 2013; Gesiarz and Crockett, 2015).

### 2.2. Major challenges

The combination of deep learning and RL used in the state-of-the-art RL algorithms has shown dramatic success in both theory and practice. Nonetheless, the computational principle of deep RL is still different from the way human RL works. Let us recall Bellman's optimality equation (Equation 1). The optimality of the policy is in principle determined by the expected amount of *long-term cumulative rewards* over a *state-action-state transition probability distribution*, each of which we call *rewards* and the *model* of the environment, respectively.

A majority of RL agents, including state-of-the-art RL algorithms, usually incorporate the MF RL principle. Here, the state-action-state transition probability is often replaced with an empirical sampling from the environment; it does not require an explicit representation of the model of the environment. Recent works such as *Muesli* (Hessel et al., 2021) exhibited the capacity to learn a model of sophisticated state representation as opposed to this, but they are mostly limited to show the planning capacity on top of the model learned i.e., learning from a simulation of possible futures using a model of an environment. This is likely to fail to demonstrate the human's capability to introspect their thought process (MB RL), and to account for the human's behavioral flexibility in arbitrating between MF and MB, the characteristics called “meta-control.”

It thus lacks the ability to develop goal-directed policies, making itself less flexible although RL algorithms are a simple and effective way to find and explore better policies. For example, a major number of classical MF RL agents requires all new learning when a new goal, such as “find a piece of cheese instead of a cup of water,” “achieve the lowest possible score,” or “achieve a goal without embarrassing your opponent,” is established (Lake et al., 2017). This is time-consuming and inefficient; it requires a lot of experience (resampling from the environment) because an MF RL agent mostly is likely to rely on the retrospective learning principle i.e., learning from past experience. This in consequence makes the training of RL agents slower and less flexible, which has been a challenge from a computational point of view.

Due to the perceived shortcomings of MF RL approaches, a growing number of MB RL algorithms have been suggested (Moerland et al., 2020) as neuroscientific findings on MB RL agents have been shown to achieve goal-directed behavioral adaptations (Doya et al., 2002; Lee et al., 2014). MF RL algorithms require a massive amount of experience to learn. This in turn leads to a significant diminution in their ability to rapidly adapt to dynamic environments where a context and its associated required tasks are frequently changed. As widely known, MB RL algorithms appear to have many potential gains to this end, such as sample efficiency, or fast adaptation to environmental changes (Daw et al., 2011; Moerland et al., 2020).

However, it is not entirely clear that MB RL is always superior to MF RL in sample efficiency, particularly in a single task. It is still in doubt that the time for learning a model and planning with the model is quicker than that for learning an optimal policy directly from the episodes under this circumstance. In addition, it is arguable whether MB RL is always better than MF RL with respect to its fast adaptation ability (Kim and Lee, 2022; Wan et al., 2022). For instance, a recent MF RL algorithm was able to achieve zero-shot learning to new goals that it never experienced during learning (Stooke et al., 2021). This algorithm was clearly MF RL, since it does not possess any model learning or planning capacity.

There is mounting evidence in decision neuroscience that has led to clarification of the principles of how the brain solves the aforementioned issues. One line of evidence suggests that the human brain employs not only MF RL but also MB RL. Other evidence indicates that the brain has the ability to learn from a few or even a single observation(s) in a process dubbed “one-shot inference” (Lee et al., 2015; Garcia and Bruna, 2018). Specifically, the human brain is engaged in determining when to learn incrementally or rapidly to make inferences regarding the state of the world. This process can be extremely useful in guiding an agent's behavior during learning (Meyniel et al., 2015), in deciding whether to explore a new alternative or pursue a currently available option (Daw et al., 2006; Badre et al., 2012), or in evaluating an alternative course of action (Boorman et al., 2009). In the following section, we will investigate how the human brain implements MF/MB RL itself and one-shot inference to guide MB RL, and how these different functional units are controlled in the brain.

## 3. Computational principles of RL in the human brain

It is widely accepted that human behavior is accounted for by two different behavior control strategies: stimulus-driven and goal-directed behavior control (for a more extensive review on these strategies, see O'Doherty et al., 2017). Historically, the brain has been thought to exert stimulus-driven behavior control (Thorndike, 1898). According to this theory, a biological agent exhibits habitual response patterns that are highly insensitive to changes in the consequences of its actions (Thibodeau et al., 1992). This has been contrasted with the idea of goal-directed behavior control, wherein deliberative actions are motivated by a specific goal (Tolman, 1948; Valentin et al., 2007).

Each strategy provides a different complementary solution considering accuracy, speed, and cognitive load (O'Doherty et al., 2017). Goal-directed behavior control allows humans to pursue adaptation to environmental changes without re-experiencing (or re-sampling) (Tolman, 1948). However, it is cognitively demanding and therefore slow. In contrast, stimulus-driven behavior control is cognitively productive, automatic, and fast despite being fragile in a volatile environment (O'Doherty et al., 2017). It appears that humans use specific principles to determine the dominating type of control to guide behavior in different contexts (Dickinson et al., 1983).

The above behavioral findings highlighting the two contrasting behavior control strategies beg the question of whether and how the human brain implements respective RL strategies (Doya, 1999; Daw et al., 2005; O'Doherty et al., 2015). As Daw et al. (2005) have proposed, the two distinct types of RL (MF and MB RL) guide human behavior, and can account for habitual and goal-directed behavior control, respectively. In the following section, we will focus on exploring the neural correlates of MB and MF RL in order to better understand the computational principles underlying RL in humans.

### 3.1. Neural correlates of RL

Animals learn to survive by making choices that lead to the receipt of rewards (e.g., food or water) and avoidance of penalties (e.g., sickness or death). In doing so, the animal should be able to estimate the value of each environmental option. This ecological conception has motivated research on the neural representations of value signals in the brain (Camerer et al., 2005; Padoa-Schioppa and Assad, 2006; Glimcher and Fehr, 2013; Juechems et al., 2017). Such investigations indicate that the value signals are found in several brain regions including the amygdala, orbitofrontal cortex, ventromedial prefrontal cortex, and ventral and dorsal striatum (Saez et al., 2015; O'Doherty et al., 2017), as well as the parietal and supplementary motor cortices (Hampton et al., 2006; Gläscher et al., 2008; Boorman et al., 2009).

**Figure 1:**
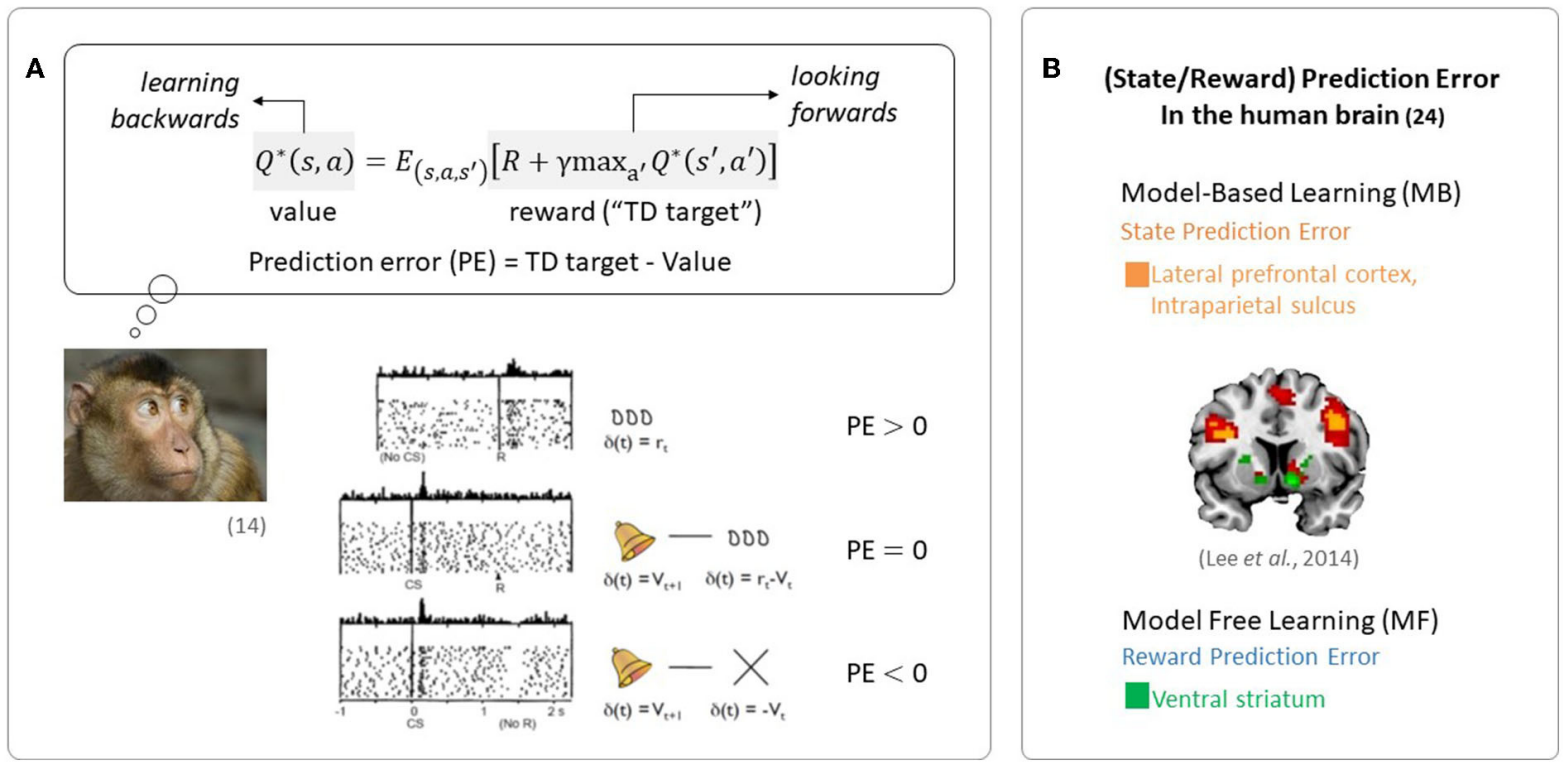
Evidence of RL in the brain. **(A)** Midbrain dopamine neurons encode information regarding the discrepancy between a predicted outcome and the actual outcome, an essential component in regulating RL (Figures are adapted from Schultz et al., 1997). **(B)** Neural evidence for the use of prediction errors in the human brain: state prediction error and reward prediction error (the figure was taken from Lee et al., 2014).

The quantity representing the discrepancy between predicted future rewards and actual rewards, called a reward prediction error (RPE) signal, is required to update the value signal. The reward prediction error signal encodes the phasic activity of dopamine neurons, as seen in Figure 1A (Schultz et al., 1997).

Since the reward prediction error plays a key role in RL, the RL framework has been used in a wide range of neuroscience disciplines. In particular, RL has been used to explain the computational functions of neuromodulators such as dopamine, acetylcholine, and serotonin (Sutton, 1988; Sutton and Barto, 1998). Phasic firing patterns of dopaminergic neurons reflect the characteristics of temporal difference prediction error in humans (Niv, 2009). Earlier studies have reported that dopaminergic neurons convey information regarding current events and the predictive value of the current state, and that the circuitry involving dopaminergic nuclei uses this information to compute a temporal difference-style reward prediction error (Christoph et al., 1986; Floresco et al., 2003; Nakahara et al., 2004; Geisler and Zahm, 2005; Matsumoto and Hikosaka, 2007). Human neuroimaging studies have also reported evidence for the presence of temporal difference prediction error signals in dopamine neurons (Glimcher, 2011; Lee et al., 2012) and reward/state prediction errors in the human brain, as shown in Figure 1B (Lee et al., 2014). In summary, these findings indicate that MF RL, including the temporal difference model, is by far the most appropriate theoretical principle to explain how animals, including humans, learn to survive.

### 3.2. Trade-off between prediction performance and computational costs: Model-based and model-free RL

Typical MF RL algorithms can successfully account for choice patterns in simple decision-making tasks. However, they fail to explain the choice patterns in multi-stage Markov decision tasks (Gläscher et al., 2010; Lee et al., 2014). There is thus a need to test the hypothesis that additional type(s) of RL strategies may be used at different time points (Dickinson et al., 1983).

Behavioral evidence indicates that there are at least two types of behavior manifesting at different time points. For example, in the early stage of training, an agent is likely to select an action based on predicted outcomes, while later on, action is elicited by a prior antecedent stimulus. The former and latter are called goal-directed and habitual behavior, respectively. Accumulating evidence supports the existence of separate neural substrates guiding these two types of behavior (Dickinson et al., 1983; Dayan and Berridge, 2014; Nasser et al., 2015; O'Doherty et al., 2017).

**Figure 2:**
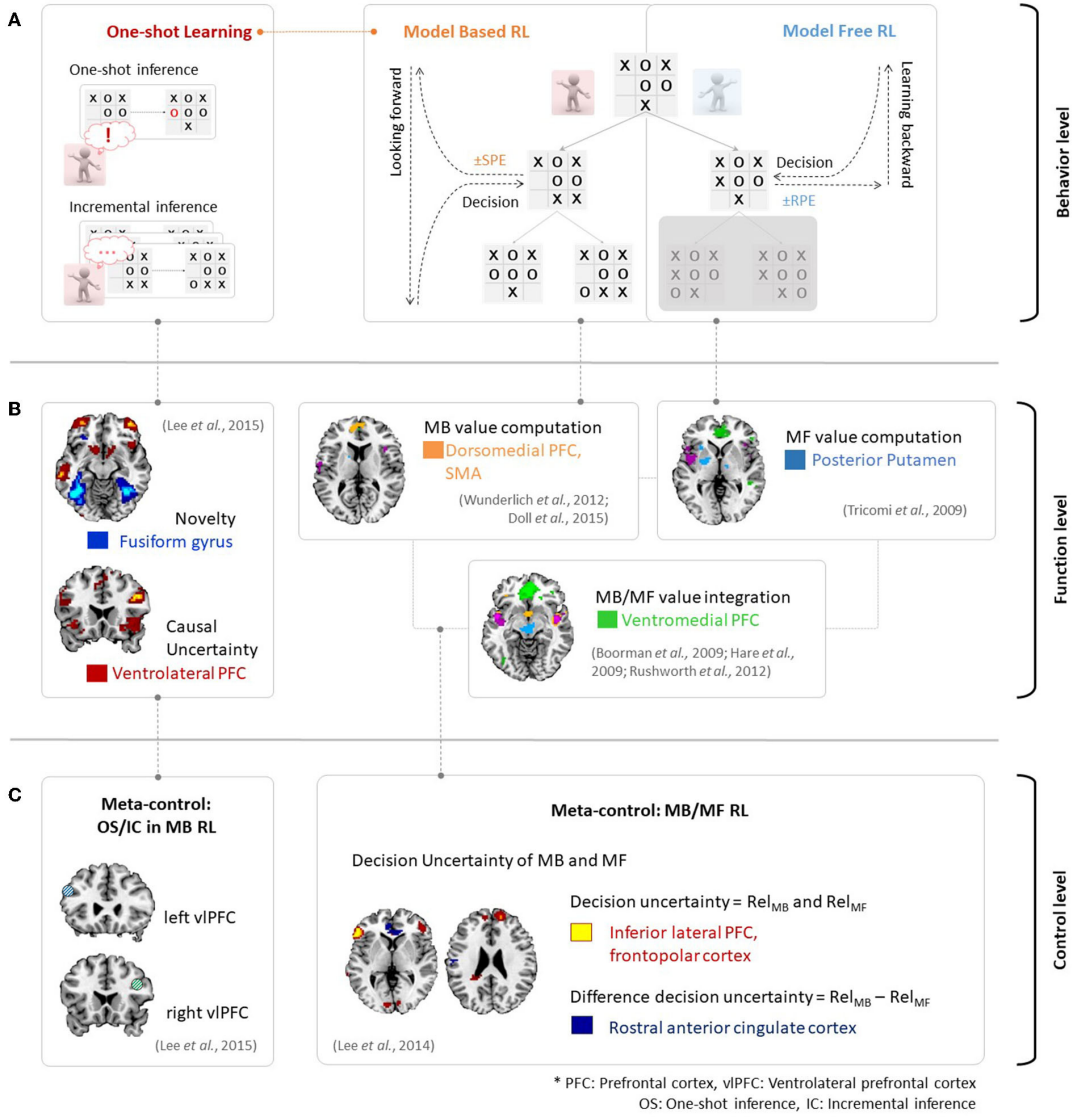
**(A)** Behavioral evidence for MB/MF RL and incremental/one-shot inference in MB RL in the context of the game of Tic-Tac-Toe. **(B)** Neural evidence for MB/MF RL and one-shot inference. Figures are adapted from Wunderlich et al. (2012); Doll et al. (2015) for MB value, Tricomi et al. (2009) for MF value, Boorman et al. (2009); Hare et al. (2009); Rushworth et al. (2012) for MB/MF value integration and Lee et al. (2015) for one-shot inference. **(C)** Neural evidence of meta-control over MB and MF RL (right figure is adapted from Lee et al., 2014), and incremental and oneshot-inference (left figure is adapted from Lee et al., 2015).

Figure 2A provides an example of the above phenomena in the context of the strategic game Tic-Tac-Toe. An MF RL agent would attempt to choose the next strategy so as to win the game and is in favor of maximizing the value. Here, the MF RL (right panel in Figure 2A) would update the value *via* sampling without consideration of the model of the game. Such action patterns associated with habitual behavior are accounted for by MF RL algorithms, which learn the values of actions based on reward prediction errors in a process of backward learning. In contrast, an MB RL agent (middle panel in Figure 2A) would first learn a model for the game (i.e., state-action-state transition probability). It would then decide on an option to win the game. The action patterns associated with this goal-directed behavior are accounted for by MB RL, which uses state-prediction errors to learn the values of actions online by combining information regarding the estimated outcome and the learned model of the environment. Therefore, an exploration stage to model an environment (e.g., a few more plays with the opponent in this case) is necessary in this context.

The major distinction between MB and MF RL is the assumption that the agent uses the knowledge of the environment to update action values as described above. For example, the MB learner computes the expected future outcome using a state-action-state transition probability distribution, whereas the MF learner does not rely on the availability of a perfect state-transition model. Neural evidence supports this assumption, as shown in Figures 2A, B. Based on the prediction error signals described in Figure 1B, a neural mechanism would process value signals from several brain regions in human RL. These brain regions include the dorsomedial prefrontal cortex, which encodes an MB value (Wunderlich et al., 2012; Doll et al., 2015), the posterior putamen, which encodes an MF value (Tricomi et al., 2009), and the ventromedial prefrontal cortex, which integrates MB and MF values (Boorman et al., 2009; Hare et al., 2009; Rushworth et al., 2012).

This computational distinction suggests that there is an inevitable compromise between the two strategies. MB RL provides more accurate predictions than MF RL in general, though both processes converge upon an optimal behavior strategy. As a result, performance differences between the two strategies diminish over time. Nevertheless, MB RL is computationally heavier than its counterpart. This indicates that there is a trade-off between prediction performance and computational costs.

### 3.3. Trade-off between prediction performance and time constraints: Incremental and one-shot learning

It is not surprising that RL agents require a sufficient number of experiences to fully learn causal relationships in the presence of different environmental factors. This is the basic principle underlying incremental inference. In this case, the agents gradually learn through trial and error to identify stimuli leading to particular consequences. There has been substantial progress in understanding the computational mechanism underlying incremental inference. Various algorithms, such as the Rescorla-Wagner rule (Rescorla and Wagner, 1972), the probabilistic contrast model (Jenkins and Ward, 1965), the associative learning model (Pearce and Hall, 1980; McLaren and Mackintosh, 2000), and Bayesian causal inference (Griffiths and Tenenbaum, 2009; Holyoak et al., 2010; Carroll et al., 2011) provide computational accounts for the behavioral characteristics associated with incremental inference. Note that an MB RL agent would gradually learn about the model of the environment if the incremental inference strategy is used.

Unlike in incremental inference, the agent sometimes learns the associations very rapidly after a single exhibition of a novel event never experienced before. This is called “one-shot” inference. This ability has been demonstrated in animal learning (Moore and Sellen, 2006; Schippers and Van Lange, 2006; Garety et al., 2011; Moutoussis et al., 2011) and object categorization (Fei-Fei et al., 2006). Although the distinctive case of one-shot inference has been relatively well-discussed in behavioral studies (Moore and Sellen, 2006; Garety et al., 2011; Moutoussis et al., 2011), its computational mechanism has received scant attention. Lee et al. (2015) investigated the computational and neural mechanisms underlying one-shot inference. They presented evidence indicating that the level of uncertainty regarding “cause-effect” relationships mediates the transition between incremental and one-shot inference. For example, more causal uncertainty leads to the assignment of a higher learning rate to a stimulus. This in turn helps resolve uncertainty and facilitates very rapid one-shot inference. This explains when and how one-shot inference occurs in preference to incremental inference, and how the brain is able to switch between the two learning strategies.

Figure 2 provides an example of the behavioral and neural evidence regarding incremental and one-shot learning in MB RL. Incremental inference (bottom-left panel in Figure 2A) usually requires considerable experience or frequent exposure to a cause-effect pairing to learn the causal relationship between the two events. On the other hand, one-shot inference learning (top-left panel in Figure 2A) requires only a single exposure to a cause-effect pairing or a single experience. In the Tic-Tac-Toe game context, a player may establish the winning strategy based on the number of game plays when he or she is using incremental inference. However, a player may also establish the winning strategy based on a single novel experience when using one-shot inference. Recent findings proposed by Lee et al. (2015) describe the neural activity associated with one-shot learning. As seen in Figure 2B, the ventrolateral prefrontal cortex (vlPFC) encodes causal uncertainty signals and the fusiform gyrus encodes the novelty of a given cause-effect pair. The fusiform gyrus then plays a crucial role in the implementation of switching control between incremental and one-shot inference.

Note that there is a trade-off issue here as well. For instance, an RL agent that learns incrementally provides more reliable predictions but is slower than one based on one-shot inference. Determining the strategy that the agent pursues would depend on prediction performance and time constraints.

### 3.4. Prefrontal meta-control to resolve the performance-efficiency-speed trade-off

As described earlier, the brain exerts control over behavior using multiple complementary strategies: (i) MB and MF RL, and (ii) incremental and one-shot learning. The former addresses the trade-off between prediction performance and computational efficiency, while the latter addressed the trade-off between prediction performance and learning speed.

These findings beg the question of whether the brain implements a principled policy to arbitrate between the two sets of strategies. Earlier theoretical work hypothesized that there exists a brain region that determines the amount of influence of each strategy on behavior (Daw et al., 2005). Subsequent studies have found evidence for the existence of such a mechanism in the human brain: the arbitration between MB and MF RL (Lee et al., 2014), and that between incremental and one-shot learning (Lee et al., 2015).

Such arbitration processes are predominantly found in the ventrolateral prefrontal cortex (Lee et al., 2014, 2015), as seen in Figure 2C. On the one hand, the ventrolateral prefrontal cortex computes the decision uncertainty in MB and MF RL while taking into account the prediction error (reward prediction error in MF RL and state prediction error in MB RL). This results in the model choice probability (*P*_*MB*_). The ventrolateral prefrontal cortex chooses the more reliable system (either MF or MB) depending on *P*_*MB*_. This would in turn control the behavior, as appropriate given the situation (right panel in Figure 2C). On the other hand, the ventrolateral prefrontal cortex is also involved in the choice of the mode of inference depending upon the degree of functional coupling between the ventrolateral prefrontal cortex and hippocampus. Specifically, the degree of functional coupling increases when one-shot inference is predicted to occur and decreases when incremental inference is predicted in the hippocampus.

The main finding of the above studies is that the key variable for arbitration is uncertainty in the prediction performance for each strategy. For example, let us assume that the MF RL agent has recently indicated high reward prediction errors, while the MB RL system has indicated low state prediction errors simultaneously at a particular moment. This would imply that the MF agent is less reliable while the MB system (i.e., goal-directed system) is warranted at this moment. In this situation, the behavioral policy would be influenced by the MB system while the brain reduces the influence of the MF system. However, it is noted that MB RL is a computationally expensive process, so the brain seems to resort to the MF RL in situations wherein the agent does not gain considerable benefit from learning the environment, such as when the environment is sufficiently stable for MF RL to make precise predictions, or when it is extremely unstable to the extent that the predictions of MB RL become less reliable than those of MF RL.

The same principle applies to the arbitration between incremental and one-shot learning. When the uncertainty in the estimated cause-effect relationships is high, the brain tends to transition to one-shot learning by increasing its learning rate. This would help the agent quickly resolve uncertainty in predicting outcomes. However, when the agent is equally uncertain about all possible causal relationships, the brain seems to resort to incremental learning. In summary, one of the important goals of RL in the human brain is to reduce the total amount of uncertainty in prediction performance. In doing so, it naturally resolves the trade-offs among prediction performance, computational efficiency, and learning speed. When using the above approach (based on the accuracy of predictions), the cognitive effort required for behavior control (FitzGerald et al., 2014) and the potential cumulative benefits inferred by an MB strategy (Pezzulo et al., 2013; Shenhav et al., 2013) are often taken into account in the meta-control of MB and MF RL.

### 3.5. Multi-agent model-based and model-free RL

New issues arise when you have a system consisting of multiple interacting agents. There are two basic cases, the cooperative case where agents have fully aligned objectives. This case is important for many applications (Claus and Boutilier, 1998; Panait and Luke, 2005). The other case, more common in nature, is called non-cooperative. Interactions in non-cooperative situations may be either fully competitive (zero-sum in the language of game theory) or partially competitive.

AlphaGo (Silver et al., 2016), an agent that defeats top human Go players is arguably the most successful example to date of an artificial system that combines MB and MF mechanisms. However, it does not attempt to combine them in a biologically-plausible manner. AlphaZero works by alternating learning a policy network by MF RL and improving it by Monte-Carlo tree search (an MB RL method) (Silver et al., 2017a,b). One reason MB methods work so well on board games but not in other domains is that a perfect model is available in these cases. The rules of the game are a complete description of the one-step transition function, and they are assumed to be known *a priori* and perfectly. This assumption is true of board games like Go and Chess but it does not even hold for games of imperfect information like Poker, much less for complex real-world environments.

Many multi-agent interactions that have been important in human evolution have partially competitive and partially aligned incentives. In particular, there are social dilemma situations. These are situations where individuals profit from acting selfishly, but the group as a whole would do better if all individuals curbed their egoism and instead acted toward the common good (Kollock, 1998). Famous social dilemmas arise in cases where there are resources that have properties making it difficult for any individual to exclude others from accessing them. For example, if all community members may access a common fishery, each individual is expected to catch as many fish as they can since they each gain from every additional fish they catch. But if all behave this way then the stock of still uncaught fish will be depleted too quickly causing the fishery to decline in productivity. This scenario and others like it have been called the tragedy of the commons (Hardin, 1968). Diverse theories of human evolution agree that navigating social dilemmas like these have been critical, especially as we have become more and more of an obligate cultural species, unable to survive even in our own ancestral ecological niche (hunting and gathering) without significant cooperation (Henrich, 2015).

There is a large classical literature concerned with agents that cooperate in abstracted matrix game models of social dilemma situations like iterated prisoner's dilemma (Rapoport et al., 1965; Axelrod and Hamilton, 1981). More recently, several algorithms have been described that achieve cooperation in more complex temporally extended settings called Markov games. Formally this setting is a straightforward generalization of Markov decision processes to multiple players (Littman, 1994). Some recent algorithms can be seen as MB (Kleiman-Weiner et al., 2016; Lerer and Peysakhovich, 2017), like in AlphaGo, the agent is assumed to have a perfect model of the rules of the game. These algorithms work in two stages, first there is a “planning” stage where the agent simulates a large number of games with itself and learns separate cooperation and defection policies from them by applying standard MF RL methods toward both selfish and cooperative objectives independently. Then in the execution phase, a tit-for-tat policy is constructed and applied using the previously learned cooperate and defect policies.

Some recent algorithms have sought to break down the strict separation between planning and execution stages and instead work in a fully on-line manner. One model-based example is the LOLA algorithm (Foerster et al., 2018). In addition to assuming perfect knowledge of the game rules, this model also assumes that agents can differentiate through one another's learning process. That is, it assumes that all agents implement a policy gradient learning algorithm. This allows agents to “learn to teach” since they can isolate the effects of their actions on the learning of others. It is possible that learned models for the environment and the learning updates of other players could be substituted in the process, but this has not yet been shown convincingly.

Another line of research on resolving multi-agent social dilemmas is based on MF RL. It drops the need for assuming perfect knowledge of the game rules (a perfect model) and works most naturally in the standard fully online setting (Leibo et al., 2017; Perolat et al., 2017). Considerable evidence from behavioral economics shows that humans have inequity-averse social preferences (Fehr and Schmidt, 1999; Henrich et al., 2001; McAuliffe et al., 2017). One algorithm in this class, proposed first for matrix games (Gintis, 2000; De Jong et al., 2008), and later extended to Markox games (Hughes et al., 2018), modifies standard MF RL to use the following inequity-averse reward function.

Let *r*_1_, …, *r*_*N*_ be the payoffs achieved by each of *N* players. Each agent receives the subjective reward


(2) $$\begin{array}{l} U_{i}(r_{i},\ldots r_{N})=r_{i}\\ \;-\frac{\alpha_{i}}{N-1}\sum_{j\neq i}\max\left(r_{j}-r_{i},0\right)\\ \;-\frac{\beta_{i}}{N-1}\sum_{j\neq i}\max\left(r_{i}-r_{j},0\right)\text{​}, \end{array}$$

The alpha parameter controls disadvantageous inequity aversion (“envy”) and the beta parameter controls advantageous inequity aversion (“guilt”). Simulations of agents with high beta parameters show that they are able to discover cooperative equilibria more easily than selfish agents since individuals are disincentivized from improving their policy in directions from which they benefit at the expense of the rest of the group. In addition, agents with high alpha parameters sometimes appear to act as “police”, punishing anti-social behavior in other agents, thereby disincentivizing defection and promoting cooperative outcomes (Hughes et al., 2018).

Disadvantageous inequity aversion is thought to be present in other species while advantageous inequity aversion may be uniquely human (McAuliffe et al., 2017). Both depend on the same neural circuity for valuation that support non-social decision-making (Fehr and Camerer, 2007). One especially relevant study found that activity in the ventral striatum and ventromedial prefrontal cortex were significantly affected by both advantageous and disadvantageous inequity (Tricomi et al., 2010).

## 4. Potential research directions

Here, we show that the convergence of computer science and decision neuroscience can extend our understanding of how the human brain implements RL. Given the evidence thus far, human RL appears to utilize not only multiple systems, but also a flexible meta-control mechanism to select among them.

**Figure 3:**
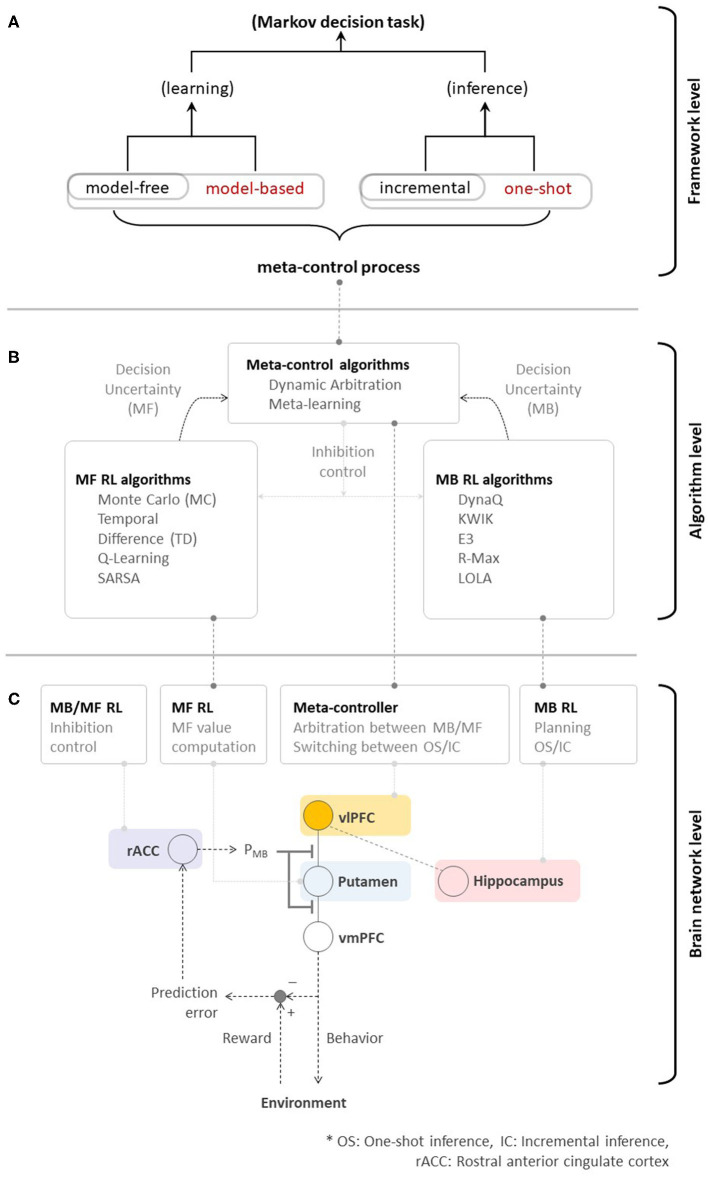
**(A)** Toward high performance, memory-efficient, and fast reinforcement learning—meta-control over MB/MF RL, and incremental/one-shot inference could ultimately accelerate the overall performance of algorithmic RL. **(B)** Neural evidence supporting algorithmic RL. **(C)** A brain network for human RL.

**Figure 4:**
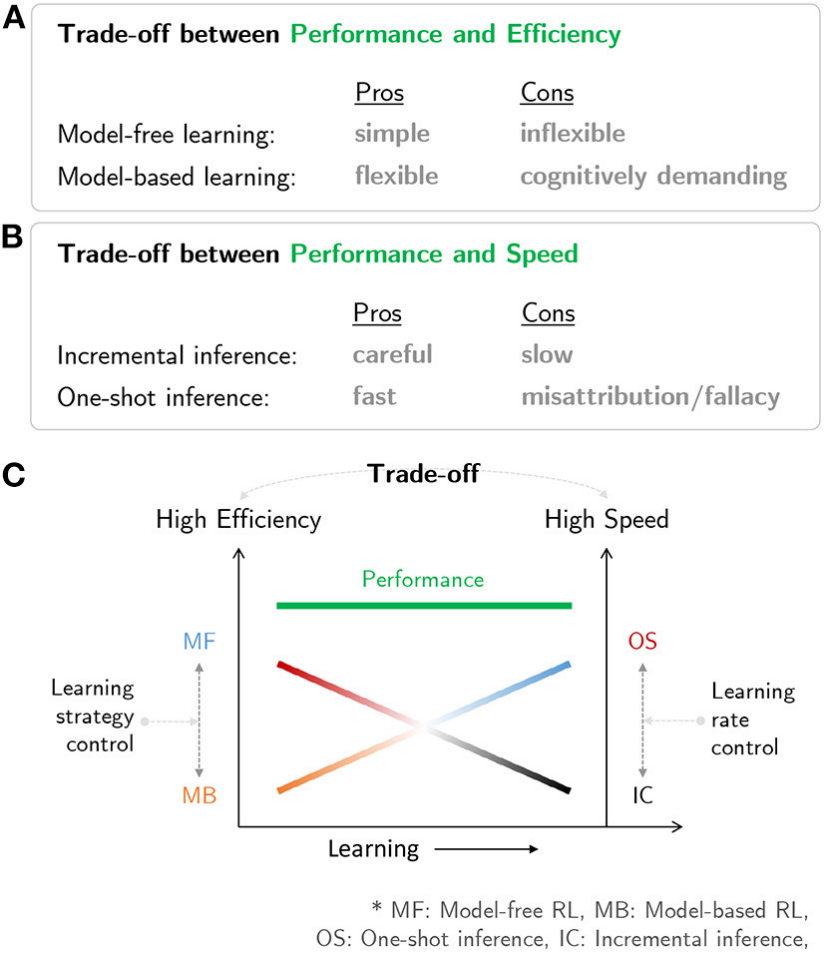
Characteristics of multiple systems in human RL: **(A)** Trade-off between performance and efficiency in MB/MF systems. **(B)** Trade-off between performance and speed in one-shot and incremental inference systems. **(C)** A neural mechanism in the human mind that resolves the trade-off issues among prediction performance, efficiency, and speed.

Figure 3C is a schematic diagram summarizing the brain network for human RL. The multiple systems facilitating human RL comprise (i) an MB system that is flexible but cognitively demanding, (ii) an MF system that is simple but inflexible, (iii) an incremental inference system that is careful while learning but slow, and (iv) a one-shot inference system that is fast in learning but has the potential to misattribute (Figures 4A, B). Based on the characteristics of the multiple systems, human RL flexibly chooses the most appropriate system while taking into account performance, efficiency, and speed. When situated in a completely new environment, meta-control accentuates speed and performance: the human RL system learns about the environment as fast as possible using the one-shot inference system while utilizing the cognitively demanding MB system to maximize performance with (relatively) lower confidence based on prior knowledge. In other situations, meta-control prioritizes efficiency and speed: the human RL system uses simple and efficient MF systems to maximize performance with accurate knowledge that is carefully constructed over time using the incremental inference system (Figure 4C).

This principle of human RL could shed light on the manner in which fundamental issues in engineering are resolved with a focus on the trade-offs among performance, efficiency, and speed, particularly in the design of artificial agents and their embodiments, robots. As highlighted in the game example, the discordance between human RL and algorithmic RL is seen in Figures 3B, C lies in the existence of the ability to flexibly control the agent's behavior in the face of dynamic changes in the environment (e.g., goals and rewards). Therefore, the principle of human RL will fuel the advent of embodied algorithms enabling RL agents to show super-human or super-artificial intelligence performance.

Of course, previous studies focusing on individual RL systems have substantially contributed to the birth of various RL algorithms. As seen in Figure 3B, MF RL algorithms, such as Monte-Carlo methods (Barto and Duff, 1994; Singh and Sutton, 1996), TD methods (Sutton, 1988), Q-Learning (Watkins and Dayan, 1992), and SARSA (Rummery and Niranjan, 1994; Sutton, 1995), share a resemblance to the function of the striatal system (that guides habitual behavior). MB RL algorithms, such as DynaQ (Sutton and Barto, 1998), KWIK (Li et al., 2008), E3 (Kearns and Singh, 2002), R-Max (Brafman and Tennenholtz, 2002), and Learning with Opponent-Learning Awareness (Foerster et al., 2018), have a potential to arguably implement the function of the prefrontal cortex (that guides goal-directed behavior). In the human brain, the ventrolateral prefrontal cortex plays an important role in the meta-control among multiple systems. Inspired by this process, dynamic arbitration (Lee et al., 2014) algorithm has introduced the preliminary implementation of meta-control while meta-learning (Wang et al., 2016, 2018) emulated similar behavioral characteristics based on the MF RL approach. While using a context-aware, model-based RL to control the model-free to manage low-level skills is another viable solution of meta-control (Lee et al., 2009; Kulkarni et al., 2016; Hamrick et al., 2017), it doesn't appear to match with the prefrontal RL strategy (Lee et al., 2014; Wang et al., 2018; O'Doherty et al., 2021; Correa et al., 2022). A deeper investigation of the theory of RL in the human brain has not only inspired, but also justified the design of advanced RL algorithms (e.g., actor-critic, Barto et al., 1983) in addition to such progress. Rapid advances in deep neural network design enable the acceleration of such developments.

Such dramatic advances have resulted in the emergence of new unresolved issues. As discussed in Section 1, the fundamental principle of advanced algorithmic RLs is still (somewhat) far from that used in human RL. Figure 3B provides a good simple example highlighting this issue. While individual learning systems have been well-studied, the system(s) working at the meta-level (ventrolateral prefrontal cortex in this case), which is seen as a means to drive the optimal learning strategy subject to changes in goals and environments, has not been carefully taken into account.

We expect that an RL agent lacking the meta-control ability to integrate multiple learning strategies may not guarantee reliable prediction and adaptation performance. For example, an agent with an MB RL bias would start to make incorrect predictions when the measurement becomes noisy (i.e., due to high measurement noise or increasing uncertainty in the environmental structure) despite consuming a large amount of computing resources. Another possible scenario is that an RL agent with a fixed high learning rate (one-shot learning) may become unstable in learning about the environmental structure.

In practice, little investigation of the unified framework approach at the algorithmic level during RL has been performed. This is despite the fact that in the past few years, the neuroscientific community has made progress in understanding the neural basis of human intelligence. At the neuroscience level, progress has been made in identifying the neural circuit engaged during learning, as described in this paper. Converging evidence implicates the frontal pole as the core element of a second-order network able to read out the uncertainty associated with computations performed by other cortical circuits (Fleming et al., 2010; De Martino et al., 2013). Nevertheless, the lack of a clear algorithmic description of how meta-control appraisal interacts with learning has severely limited progress in our understanding of how striatal RL systems and the prefrontal meta-controller interact in an integrated single framework.

We firmly believe that the integration of new findings regarding MB/MF RL, and the use of rapid and slow RL in a single framework (as seen in Figure 3A) would be a natural resolution to the problems described above, as this is what our brains perform in daily life. First, supplementing MF RL with a functional module encapsulating MB RL would enable goal-directed decision-making based on a model of the environment. It will also enable rapid action selection to achieve a goal in a dynamic environment. Second, supplementing an incremental learner with a one-shot learning module would ensure that RL agents would perform rapid learning based on information from a small number of episodes. Finally, implementing meta-control on these disparate learning strategies would afford us the leverage to rapidly achieve high prediction performance with a minimal loss of computational costs. Our view is supported by the recent evidence showing that in humans, the engagement of model-based RL mitigates the risk of assigning credit to outcome-irrelevant cues (Shahar et al., 2019). This result highlights the necessity of a control mechanism to determine when the MB system should override the MF system.

It is also noted that the meta-control has a great potential for dealing with conflicting demands in multiple agent learning, such as competition v.s. cooperation or envy v.s. guilt. As recent studies demonstrate that the MB and MF RL can provide pragmatic solutions for diverse social dilemma problems, implementing meta-control would also create a possibility for optimizing the performance of multi-agent learning systems. For example, such principles can be used to deal with a competition-cooperation issue in a smart home system in which multiple robot agents and human users interact with each other, or possibly to deal with an envy-guilt dilemma in social networks or online multiplayer games.

## 5. Conclusion

Deeply rooted in interdisciplinary research, including computer science, cognitive psychology, and decision neuroscience, reinforcement learning theories provide fundamental learning principles used to solve various real-world optimal control problems. In this paper, we reviewed a computational reinforcement learning theory, as well as its applications and challenges. We then discussed how the brain may perform analogous kinds of learning. We discussed MB/MF RL and one-shot/incremental inference, as well as meta-control over multiple learning strategies and discussed the implications of MB and MF RL in problems involving multiple interacting agents. We believe that the integrated conceptual framework incorporating these functions will lead to a major breakthrough in RL algorithm design where the trade-offs among prediction performance, computational costs, and time constraints are resolved.

## Author contributions

JHL and SL contributed to the conception and design of the study. SA prepared the first draft of the figures. JHL wrote the first draft of the manuscript. JHL, JZL, and SL wrote sections of the manuscript. All authors contributed to manuscript revision, read, and approved the submitted version.
